# Discerning ADHD from comorbid Austim Spectrum Disorder, and using the Child and Adolescent approach when diagnosing and treating adults with ADHD

**DOI:** 10.1192/j.eurpsy.2025.101

**Published:** 2025-08-26

**Authors:** J. K. Suykerbuyk

**Affiliations:** Psychiatry - Rehabilitation, JKS-Miander, Antwerpen, Belgium

## Abstract

**Abstract:**

Although Autism Spectrum Disorder (ASD) is frequently reported as a comorbidity in adults with Attention Deficit Hyperactivity Disorder (ADHD), diagnosing this additional condition in clinical practice remains challenging. Missing this diagnosis can significantly impact treatment and reduce the quality of life for these adults. In child and adolescent psychiatry, attention is also given to the source(s) that influence the symptoms and complaints of ASD and ADHD, both in diagnosis and treatment.

This lecture highlights the importance of identifying the source(s) of symptoms and complaints in adult patients, with or without ADHD, in an outpatient urban setting. Better mapping of these source(s) helps distinguish the symptoms of ASD from those of ADHD, refining both diagnosis and treatment. Unsurprisingly, ASD is often not even considered a potential comorbid condition in adults. In this lecture, we share practical experiences with adult outpatients and discuss the source(s) that frequently emerge, which aid in differentiating ASD symptoms from those of ADHD.

**Disclosure of Interest:**

None Declared

